# Attributes, Quality, and Downloads of Dementia-Related Mobile Apps for Patients With Dementia and Their Caregivers: App Review and Evaluation Study

**DOI:** 10.2196/51076

**Published:** 2024-04-29

**Authors:** Tzu Han Chen, Shin-Da Lee, Wei-Fen Ma

**Affiliations:** 1 PhD Program for Health Science and Industry China Medical University Taichung Taiwan; 2 PhD Program in Healthcare Science Department of Physical Therapy China Medical University Taichung Taiwan; 3 PhD Program in Healthcare Science School of Nursing China Medical University Taichung Taiwan; 4 Department of Nursing China Medical University Hospital Taichung Taiwan

**Keywords:** app quality, caregiver, dementia, geriatrics, aging, technology, digital health, mHealth, mobile health, seniors, mobile app, patient, adoption, development, management

## Abstract

**Background:**

The adoption of mobile health (mHealth) apps among older adults (>65 years) is rapidly increasing. However, use of such apps has not been fully effective in supporting people with dementia and their caregivers in their daily lives. This is mainly attributed to the heterogeneous quality of mHealth apps, highlighting the need for improved app quality in the development of dementia-related mHealth apps.

**Objective:**

The aims of this study were (1) to assess the quality and content of mobile apps for dementia management and (2) to investigate the relationship between app quality and download numbers.

**Methods:**

We reviewed dementia-related mHealth apps available in the Google Play Store and Apple App Store in Taiwan. The identified mobile apps were stratified according to a random sampling approach and evaluated by five independent reviewers with sufficient training and proficiency in the field of mHealth and the related health care sector. App quality was scored according to the user version of the Mobile Application Rating Scale. A correlation analysis was then performed between the app quality score and number of app downloads.

**Results:**

Among the 17 apps that were evaluated, only one was specifically designed to provide dementia-related education. The mean score for the overall app quality was 3.35 (SD 0.56), with the engagement (mean 3.04, SD 0.82) and information (mean 3.14, SD 0.88) sections of the scale receiving the lowest ratings. Our analyses showed clear differences between the top three– and bottom three–rated apps, particularly in the entertainment and interest subsections of the engagement category where the ratings ranged from 1.4 to 5. The top three apps had a common feature in their interface, which included memory, attention, focus, calculation, and speed-training games, whereas the apps that received lower ratings were found to be deficient in providing adequate information. Although there was a correlation between the number of downloads (5000 or more) and app quality (t_15_=4.087, *P*<.001), this may not be a significant determinant of the app’s perceived impact.

**Conclusions:**

The quality of dementia-related mHealth apps is highly variable. In particular, our results show that the top three quality apps performed well in terms of engagement and information, and they all received more than 5000 downloads. The findings of this study are limited due to the small sample size and possibility of disregarding exceptional occurrences. Publicly available expert ratings of mobile apps could help people with dementia and their caregivers choose a quality mHealth app.

## Introduction

### Background

The global aging population is experiencing an astonishing surge, which will inevitably result in a significant rise in the prevalence of dementia [1]. Consequently, it has become crucial to identify efficacious strategies to support people affected by dementia and enhance the well-being of their caregivers [2]. In addition, numerous studies have shown that mobile health (mHealth) apps can effectively reduce medical costs and improve quality of life for middle-aged and older adults, especially after COVID-19 [3,4].

The use of technology among older adults (aged >65 years) has triggered noteworthy transformations in health care provision [5]. An area where technology has proven especially valuable is in the realm of dementia management, with mHealth apps dominating the forefront of this field [6]. In addition, the UK government has shown support for the advancement of intelligent assistive technology for individuals with dementia [7]. This includes endorsing the development of mHealth apps specifically tailored to patients with early-stage dementia and their caregivers [8]. These apps are believed to have significant potential in aiding cognitive function and facilitating self-care among those living with dementia [9].

However, the constant emergence of mHealth apps has made it challenging for both patients with dementia and their caregivers to differentiate, evaluate, and use mHealth apps that promote healthy behaviors [10,11]. Therefore, information pertaining to dementia-related mHealth apps and their functionalities should be effectively evaluated and made publicly available.

There is significant heterogeneity in the quality of dementia-related mHealth apps [12], and most studies assessing app quality have used criteria that focused on general characteristics that could be assessed without downloading or using the app itself [13,14]. Therefore, there is a need for a human-centered, multidimensional measure that includes usability components and relatively more domains to identify high-quality mHealth apps [15]. Ideally, better features and functionality would drive high-quality apps; however, efforts to identify the differences between high- and low-quality apps have been hampered by scarce research.

Moreover, the factors that contribute to the popularity of specific mHealth apps remain largely unknown, although there is some evidence of a relationship between an app’s star rating and its number of downloads [16]. However, few studies have evaluated dementia-related mHealth apps to date. Therefore, the specific metrics of app quality that are likely to be associated with a higher number of downloads remain to be identified.

### Objective

This study had several goals. The first goal was to analyze the content of mobile apps for people with dementia and their caregivers across different categories. The second goal was to assess the quality of individual apps using the user version of the Mobile Application Rating Scale (uMARS). The third objective was to perform a comparative analysis of the highest- and lowest-quality dementia-related mHealth apps, with the broader goal of establishing guidelines to facilitate future app development. Finally, the study aimed to explore the correlation between app quality and downloads. This was done to help identify the gaps in the currently available dementia-related mHealth apps and to provide recommendations for patients with dementia and their caregivers on how to select high-quality apps.

## Methods

### Search Strategy and Inclusion Criteria

**Figure 1:**
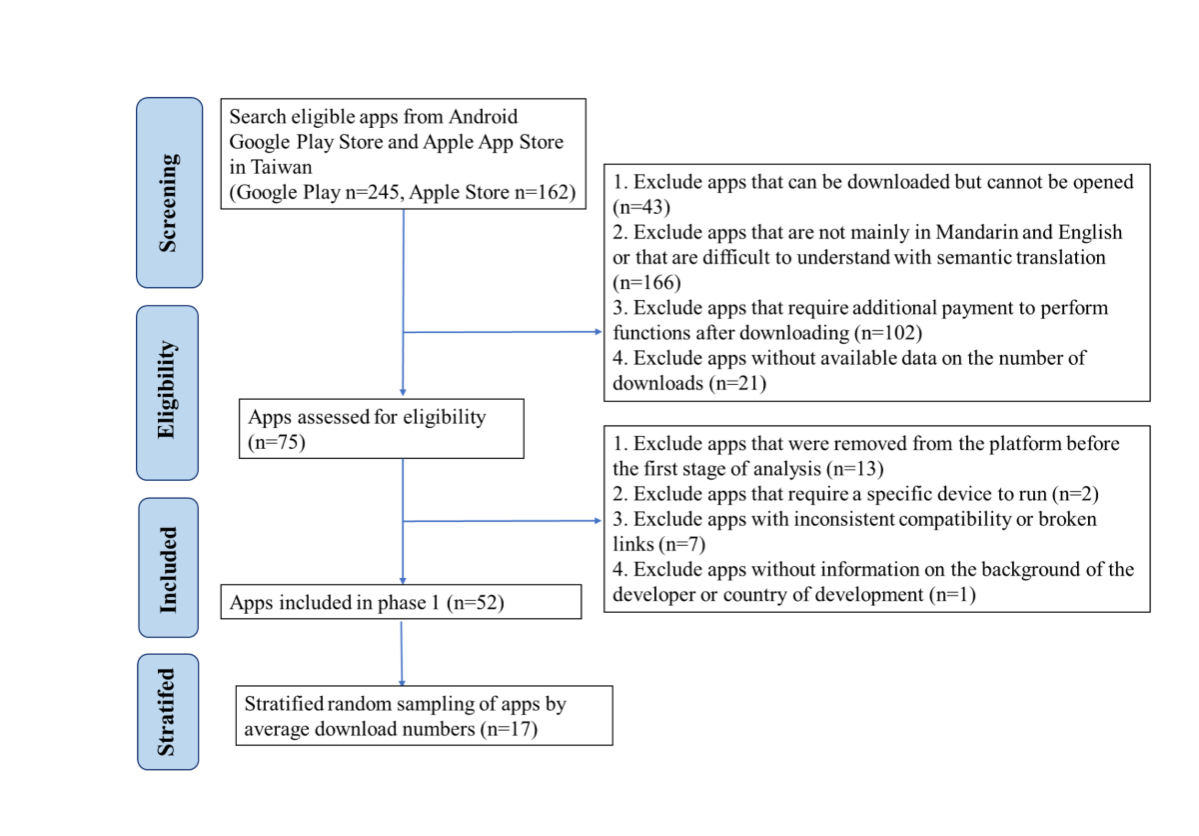
Flow diagram to illustrate the process used to identify and stratify dementia-related mobile health apps.

Apps were identified from the Taiwan Apple App Store and Google Play Store. Between July 2022 and November 2022, the following search terms (in Mandarin and English) were used in the app stores: dementia, cognitive dysfunction, dementia caregiver, Alzheimer disease, dementia care, cognitive games, and memory games. The screening criteria and process are illustrated in Figure 1.

Apps were included if they met all of the following inclusion criteria: (1) exists in the Google Play Store for Android mobile devices and the App Store for Apple mobile devices; (2) addresses daily-life topics related to neurocognitive disorders [17], and (3) was purposefully developed with the primary goal of supporting patients or caregivers (including health care workers) with the topic of mild cognitive impairment; (4) can be downloaded and used for free; (5) mainly uses Mandarin or the English version can be translated into Mandarin and is easy to understand; and (6) has been updated within the last 5 years.

### Stratified Random Sampling of Apps by Average Download Numbers

In November 2022, searches were conducted on the two platforms to find apps that met the above criteria. Of the 407 apps found, 332 were deemed ineligible after screening (Figure 1). The remaining 75 apps were thoroughly screened, resulting in 52 apps included for preliminary evaluation. Since the length of time an app has been available on a platform can affect its number of downloads, we calculated the ratio of download numbers with respect to time on the platform. Additionally, to consider uneven allocation and lack of continuity in stratification, the apps were sorted according to the ratio of downloads relative to the number of days since the release date on the platform. Thus, the average number of downloads was calculated as the total number of downloads/number of days on platform since the release date. The apps were then ranked according to the average number of downloads in ascending order, and we randomly selected 1 out of every 3 apps for a total of 17 apps that were subject to detailed quality assessment and review.

### General Characteristics and Classification

Each app was used by two authors (THC and WFM) independently. According to their content subcategory, the selected apps were categorized into four different types using the guidelines provided by the National Institute for Health and Care Excellence and the National Health Service in the United Kingdom [18,19]. Any conflicts in app classification were adjudicated by discussion between the two reviewers regarding each domain within the extraction form to reach consensus. Details on the main characteristics and comments of the included apps are provided in Multimedia Appendix 1.

### mHealth App Quality Evaluation

The uMARS is a tool that can be used to evaluate the quality of mHealth apps, including four objective subdomains: engagement, functionality, esthetics, and information. There is also a domain for subjective quality and another for perceived impact. Stoyanov et al [20] developed the uMARS in 2016, which showed excellent internal consistency (Cronbach α=0.90). The uMARS scores are rated on a 5-point Likert scale ranging from 1 (“strongly disagree”) to 5 (“strongly agree”).

The objective quality score is calculated as the average of the scores of the four dimensions. Engagement is defined as fun, interesting, customizable, interactive, and has prompts (eg, sends alerts, messages, reminders, feedback, allows sharing). Functionality refers to overall app functioning, easy to learn, navigation, flow logic, and gestural design of the app. Esthetics refers to the graphic design, overall visual appeal, color scheme, and stylistic consistency. Finally, the information domain assesses whether the app contains high-quality information (eg, text, feedback, measures, and references from a credible source). The subjective quality score reflects the rater’s personal interest in the app. The final uMARS subscale includes 6 items designed to assess the perceived impact of the app on the user’s awareness, knowledge, attitude, intention to change, help-seeking, and likelihood to change the target health behavior.

### Reviewer Recruitment and Selection

Reviewers recruited for this study were required to have a professional background in clinical treatment, the health care industry, or information engineering. Additionally, they were required to have at least 3 years of work experience in elderly health care or health technology–related fields, as well as experience using digital mobile devices. Exclusion criteria included no relevant work experience in elderly health care or health technology–related fields in the past 5 years.

Five reviewers were recruited as an interdisciplinary group of experts. The initial reviewer possessed knowledge and had experience in creating a content management system for a dementia management app. The second reviewer was a health informatics researcher with sufficient training and expertise in the relevant health care technology fields focused on dementia. The third reviewer also had extensive experience in dementia and in the mHealth industry. The fourth reviewer was a psychiatric nurse with experience in caring for older adults along with clinical experience in dementia. The final reviewer was a nurse practitioner who has been providing care for older adults and patients with dementia for over a decade.

### Evaluation Process

Each of the apps was assessed by the five reviewers and the evaluation process was conducted between December 17, 2022, and January 3, 2023. All 17 apps can be found on the Android platform; hence, the apps were reviewed when running on the same Android tablet. The experts were blinded to the download numbers, year, and country of development of the apps, and they were not allowed to discuss their assessments with each other to ensure independence in their ratings. We ensured an equal distribution of app assessments in each round by applying a ratio that took into account the download-to-time axis. Furthermore, each reviewer allocated a minimum of 30 minutes and a maximum of 1 hour to thoroughly evaluate the included apps.

### Ethical Considerations

The study received ethical approval from the ethics committee of China Medical Hospital, Taiwan, on November 8, 2022 (approval number: CMUH111-REC2-151) and was conducted according to the guidelines of the Declaration of Helsinki.

The experts in this study were not compelled to take part and had the freedom to determine their involvement. Additionally, they possessed the ability to discontinue their participation at any juncture, without being required to supply a justification for their decision.

This study utilized legally obtained publicly available information, and it was ensured that the use of information aligns with its intended public knowledge purpose. Furthermore, data collected from research and expert evaluations are stored on a hard drive and encrypted. The evaluation process was fully anonymous, with no face-to-face interactions among experts, and the evaluation of the app was a non-nominal, noninteractive, and noninvasive study. Relevant original data regarding this research will be preserved for at least 3 years after the execution period, securely locked in the principal investigator’s office cabinet.

The clinical trial protocol developed by the research institute stipulates that in the event of adverse reactions resulting in damages, China Medical University Hospital is responsible for providing compensation. Nonetheless, adverse reactions explicitly disclosed in the informed consent form signed by the experts are not eligible for compensation. This study was not covered by liability insurance and the per-expert evaluation cost was US $170.

### Statistical Analysis

The number and proportion of information displayed in the apps, including the country of app development, download number, and app type, were summarized using descriptive statistics. The uMARS scores, along with the scores for each domain and subscale, are presented as the mean and SD. The *t* test was used to examine the association between downloads and each domain of the uMARS. Statistical analyses were conducted using IBM SPSS Statistics v28 (IBM Corp). We considered *P*<.05 to indicate statistical significance in all analyses.

## Results

### App Attributes

The apps were primarily developed in the United States, and 11 out of the 17 dementia-related mobile apps were downloaded less than 5000 times. Among the 17 apps, 8 were classified as those designed to improve clinical outcomes from established treatment pathways through behavior change, and for enhancement of patient adherence and compliance with treatment; 5 were designed as standalone digital game therapeutics; 3 were classified for supporting clinical diagnosis and/or decision-making; and 1 app was primarily designed to provide disease-related education (Table 1).

**Table 1:** App characteristics (N=17).

Characteristics		Apps, n (%)	
**Country of development**			
	United Kingdom		2 (12)
	Hong Kong		1 (6)
	United States		5 (29)
	Taiwan		2 (12)
	Germany		2 (12)
	Australia		2 (12)
	India		2 (12)
	Canada		1 (6)
**Number of downloads**			
	>10		1 (6)
	>50		1 (6)
	>100		2 (12)
	>500		3 (18)
	>1000		4 (24)
	>5000		2 (12)
	>10,000		1 (6)
	>100,000		
	>1,000,000		1 (6)
	>10,000,000		1 (6)
**App type/function**			
	Improve clinical outcomes from established treatment pathways through behavior change, and enhancement of patient adherence and compliance with treatment		8 (47)
	Standalone digital game therapeutic		5 (29)
	Support clinical diagnosis or decision-making		3 (18)
	Primarily to deliver disease-related education		1 (6)

### App Quality Assessment by Interdisciplinary Experts

There was a notable level of agreement or correlation among the reviewers in their app evaluations, as indicated by the Kendall *W* statistic of 0.143, which was significant at *P*=.05.

Overall, the mean app quality score was 3.35 (SD 0.56), which ranged from 2.25 (worst-rated app) to 4.07 (best-rated app). For engagement, the mean score was 3.04 (SD 0.81). Furthermore, functionality had the highest mean score of 3.76 (SD 0.38) and showed the smallest variation in minimum and maximum scores among the apps evaluated. In other words, these apps were considered to have relatively high levels of functionality and usability by the interdisciplinary expert reviewers. The esthetic quality of the interface received a mean score of 3.45 (SD 0.65), indicating that visual design elements such as button size, icon clarity, and content arrangement were perceived as being well organized. Additionally, the information domain received a mean score of 3.14 (SD 0.88), suggesting that the presentation and accessibility of information on the screen could be improved. Multimedia Appendix 2 provides the complete details of app quality scores.

### Top Three and Bottom Three Performers in App Quality Score

The apps ranked in the top three positions according to app quality scores included Memorado Brain Games, NeuroNation-Brain Training & Brain Games, and Brain Track. The common characteristic among these apps is that their interface consists of training games focused on memory, attention, concentration, calculation, and speed. Conversely, Alz Test, American Caregiver Association, and Dementia and Me ranked in the bottom three; these three apps performed poorly on both engagement and information.

The overall scores for each item for the top three and bottom three apps are provided in Table 2. The functionality domain received the highest average ratings, particularly for gestural design, navigation, and performance. The largest discrepancies in app quality ratings between the top three and bottom three apps were found in the areas of entertainment and interest, where the scores ranged from 1.4 (worst-rated app) to 5 (best-rated app). Similarly, in the subscale of perceived impact, there was a significant difference in attitude, with ratings ranging from 1.2 (worst-rated app) to 4.2 (best-rated app).

**Table 2:** Comparison of quality between the top three– and bottom three–rated dementia-related apps.

Metric			All apps (N=17)		Memorado		NeuroNation		Brain Track		Alz Test		American Caregiver Association		Dementia and Me
Ranking (out of 17)			N/A^a^		1		2		3		15		16		17
Number of downloads			N/A		>1,000,000		>10,000,000		>5000		>1000		>100		>10
**Engagement, mean (SD)**															
	Entertain	3 (1.08)		5 (0)		4.4 (0.55)		4.2 (0.84)		2.4 (0.89)		1.8 (0.45)		1.4 (0.55)	
	Interest	3 (1.09)		5 (0)		4.2 (0.45)		4.2 (0.45)		2.4 (0.89)		1.6 (0.55)		1.4 (0.55)	
	Customize	2.67 (0.49)		3.6 (0.89)		4 (1)		3.4 (1.14)		1.8 (0.45)		2.2 (0.45)		2.6 (0.89)	
	Interactivity	2.95 (0.72)		4 (1)		4.4 (0.89)		4 (0.71)		2.4 (0.89)		2 (1.41)		2.2 (0.45)	
	Target group	3.55 (0.65)		4.4 (0.89)		4.2 (0.84)		4.2 (0.45)		2.6 (0.55)		2.6 (0.89)		2.4 (0.55)	
**Functionality, mean (SD)**															
	Performance	3.72 (0.52)		4.6 (0.55)		3.8 (0.84)		4 (0.71)		3 (1.22)		3 (1.41)		2.6 (1.34)	
	Ease of use	3.62 (0.39)		4 (1)		3.4 (0.55)		3.6 (0.55)		3.4 (0.89)		3 (1.41)		3 (1.22)	
	Navigation	3.72 (0.29)		4 (0.71)		3.6 (0.55)		3.8 (0.45)		3.6 (0.55)		3.4 (0.89)		3.2 (0.84)	
	Gestural design	3.98 (0.37)		4.6 (0.55)		4.2 (0.84)		4.2 (0.45)		3.6 (0.55)		3.2 (1.1)		3.4 (0.89)	
**Esthetics, mean (SD)**															
	Layout	3.54 (0.58)		4.4 (0.89)		4 (1)		4.4 (0.89)		2.8 (1.1)		3 (1)		3 (1)	
	Graphics	3.51 (0.53)		4 (1)		4.2 (0.84)		4.2 (0.45)		3 (1)		2.6 (0.55)		3.2 (0.84)	
	Visual appeal	3.32 (0.65)		4.2 (0.84)		4.2 (0.84)		4 (0)		2.2 (0.84)		2.4 (0.55)		2.6 (0.55)	
**Information, mean (SD)**															
	Quality	3.55 (0.61)		4.5 (0.58)		4.2 (0.45)		4.2 (0.45)		2.75 (0.5)		3.5 (1.29)		2 (1.41)	
	Quantity	3.41 (0.74)		4.5 (0.58)		4 (1)		4 (0.7)		2.5 (1.29)		3.5 (1)		2 (0)	
	Visual information	3.4 (0.66)		4.25 (0.96)		4.2 (0.45)		4.2 (0.45)		2.6 (0.58)		3 (0.82)		1.67 (0.58)	
	Credibility	3.3 (0.58)		3.75 (0.5)		3.8 (0.84)		3.4 (0.55)		3.75 (1.26)		3.25 (0.96)		1.67 (0.58)	
**App subjective quality, mean (SD)**															
	Recommend	3.2 (0.87)		4.4 (0.89)		4 (1)		4 (0.71)		2 (1)		2.2 (1.1)		1.4 (0.55)	
	Relevant	2.79 (0.88)		4.2 (0.84)		3.6 (1.14)		3.6 (0.55)		1.6 (0.55)		1.6 (0.55)		1.4 (0.55)	
	Pay	2.05 (0.7)		3.4 (0.89)		2.6 (1.52)		3.2 (0.84)		1.2 (0.45)		1 (0)		1 (0)	
	Star rating	3.05 (0.88)		4.4 (0.89)		4 (1)		4 (1)		2.2 (0.84)		2 (1)		1.4 (0.55)	
**Perceived impact, mean (SD)**															
	Awareness	3.08 (0.73)		4 (1)		3.8 (1.3)		4 (0.71)		2.2 (1.33)		2.6 (1.14)		1.2 (0.45)	
	Knowledge	3.24 (0.81)		3.2 (1.64)		3.8 (1.3)		3.8 (0.84)		2.4 (1.67)		3.2 (1.64)		1.2 (0.45)	
	Attitudes	3.04 (0.8)		4.2 (0.84)		3.6 (1.34)		4 (0.71)		2 (1.22)		2.6 (1.14)		1.2 (0.45)	
	Intention to change	3.02 (0.77)		4.2 (0.84)		3.8 (1.3)		3.6 (0.89)		2 (1.22)		2.4 (1.14)		1.4 (0.89)	
	Help seeking	3.06 (0.69)		3 (1.58)		3.6 (0.89)		3.6 (0.89)		2.4 (1.44)		2.6 (1.82)		1.2 (0.45)	
	Behavior change	3.08 (0.77)		4 (0.71)		3.8 (0.84)		3.8 (0.84)		2 (1.22)		2.4 (1.34)		1.4 (0.89)	

^a^N/A: not applicable.

### Association Between Downloads and Quality of Mobile Apps

The Connectivity in Digital Health survey of global mHealth apps reported that 55% of the apps available on the Google Play store, Apple App Store, Windows Phone Store, Amazon Appstore, and Blackberry World had fewer than 5000 total downloads [21]. Therefore, the 17 apps included in our study were divided into two subgroups based on the total number of downloads. The first subset consisted of 6 apps with more than 5000 total downloads, representing 35.3% of all apps. The mean app quality score for this subgroup was significantly higher than that of the group of apps with less than 5000 downloads (Table 3). In addition, apps with more than 5000 downloads generally had higher scores for each domain. However, neither information nor perceived impact scores were significantly correlated with the number of downloads (Table 3).

**Table 3:** Independent-samples t test of the correlation of the scores of app quality with download numbers.

uMARS^a^ domain	Score, mean (SD)			*t* statistic (*df*=15)		*P* value
	Apps downloaded >5000 times (n=6)	Apps downloaded <5000 times (n=11)				
				
App quality	3.88 (0.26)	3.06 (0.45)	4.087		<.001	
Engagement	3.95 (0.54)	2.55 (0.4)	6.054		<.001	
Functionality	4.05 (0.31)	3.6 (0.32)	2.785		*.*01	
Esthetics	4.1 (0.35)	3.1 (0.48)	4.469		*<.*001	
Information	3.43 (0.53)	2.98 (1.00)	1.210		*.*33	
Subjective quality	3.70 (0.44)	2.26 (0.40)	6.924		*<.*001	
Perceived impact	3.31 (0.50)	2.96 (0.79)	0.97		*.*35	

^a^uMARS: user version of the Mobile Application Rating Scale.

## Discussion

### Principal Findings

According to our results, there was only one included app that primarily focused on delivering dementia-related education. Furthermore, the top three quality apps were all classified as the main app type, as they all served as standalone digital game therapeutics. In general, the dementia-related mHealth apps were of moderate quality with a common characteristic of high functionality. Nonetheless, these apps exhibited poor performance in engagement and the credibility of information domain. Although we found a correlation between the number of downloads and app quality, this may not be a significant determinant of the information provided and the app’s perceived impact.

### Comparison With Prior Work

mHealth apps offer a new way to support people with dementia and their caregivers [22]. However, previous studies have pointed out that the scientific literature on the design and evaluation of web- and mobile-based health apps remains scarce [23,24]. To address this issue, our study directly assessed the app type in a practical setting and found the lack of a dementia management app that delivers disease-related education. A randomized controlled trial indicated that mHealth apps can be of educational value to patients by providing structured disease and treatment-related education; therefore, future app developers can focus on increasing the availability of this app type with educational value [25].

A previous study suggested that research collaboration between health care and software engineering experts could help advance our knowledge of app functionality and effectiveness [16]. Therefore, we established a panel of experts to obtain accurate results on the quality of currently available dementia-related mHealth apps and further identified their subjective quality and perceived impact. The pattern of high functionality and low information quality is in accordance with the findings of other studies on mobile apps designed for older adults [26]. Additionally, the inadequacy of credibility was associated with several risks, particularly in the areas of self-diagnosis, prevention, and health promotion [27].

High-quality mHealth apps offer self-management features, relaxation, recreation, and trustworthy information [28,29]. The uMARS consists of elements of usability and a broader range of areas that are used in the assessment of mHealth apps with superior quality. Notably, a consensus was reached among the reviewers in both the engagement and esthetics domains. However, there was no correlation or similarity among reviewers with respect to assessments on functionality and information of the apps. This discrepancy may be due to the different backgrounds of the reviewers [30]; health care providers may perceive the app’s information as inadequate, whereas experienced developers of dementia apps may find its functionality to be lacking.

Currently, little is known about why some health apps become popular and others do not, and researchers have demonstrated that the number of downloads on app marketplaces does not correlate with clinical utility or validity for mental health apps [31]. A study from the Netherlands and Portugal identified the predictors that might influence the number of downloads for urology apps [32]. However, there is little research on the predictors of app downloads for dementia-related mHealth apps in the PubMed database. Hence, to gain a more comprehensive understanding, the apps were stratified using a random sampling approach. Due to the different themes of mHealth apps, our study found a positive relationship between app quality and number of downloads. Finally, the download number does only seem to be a limited orientation aid for the selection of an mHealth app, and future studies should consider this aspect.

### Limitations

This study has several limitations. Initially, the search for mobile apps was conducted within a limited time frame and focused on apps that had been updated within the last 5 years. As such, the study fell short with respect to establishing causal relationships. In addition, rapidly expanding and ever-changing mobile app marketplaces are facing significant challenges in keeping pace with the dynamic landscape; hence, some of the apps evaluated in this study may have since changed or new alternatives may have been developed. Furthermore, the search for mobile apps was confined to app stores in Taiwan, which may not accurately represent app offerings in other countries due to regional disparities in developers’ decisions regarding app availability.

Previous research indicated that the cost associated with using mHealth apps acts as a major obstacle for older individuals when it comes to embracing mobile technologies [9,33]. Furthermore, a recent study discovered that 96% of mHealth apps that are accessible on the Chinese market can be downloaded without cost [34]. Consequently, one-quarter of the apps would have been overlooked if they required payment. Nonetheless, it is possible that within this group of paid apps, there may have been some high-quality apps that were unintentionally excluded from consideration.

Additionally, the stratification method represents both less popular and highly downloaded apps, mirroring real-world data [21]. However, this method resulted in a smaller sample size, which could potentially lead to some superior apps being overlooked by chance. With only 17 apps remaining for evaluation, it is possible that there may not have been sufficient statistical power to establish a significant relationship between app quality and download frequency.

Finally, to ensure a rigorous evaluation of the app content, experts from different fields were recruited to review the apps. However, the limited number of reviewers could potentially influence the results of the study, and the degree of agreement may not be strong given that the reviewers are from different disciplines and the time they allocated to evaluate each app could potentially impact the reliability of agreement.

Despite these limitations, this study helps to fill the gap in the evaluation of dementia-related mobile apps. The results can still be used to guide the selection of such apps in Taiwan and possibly other regions with similar app marketplaces, while also highlighting the need for ongoing evaluation of mobile apps for dementia care.

### Conclusions

This study set out to gain a better understanding of the characteristics, quality, and downloads of dementia-related mHealth apps. In particular, the top three quality apps were all offered as standalone digital game therapeutics, which scored well on both engagement and information quality, and received more than 5000 total downloads. Nevertheless, the findings of our investigation do not offer a comprehensive solution due to the restricted scale of the sample and the potential for overlooking extraordinary instances. Consequently, annual reviews and publicly available expert ratings of mobile apps could help people with dementia and their caregivers choose a high-quality mobile app.

## Appendix

Multimedia Appendix 1 Description, classification, and overall comments of reviewers after using the selected dementia-related mobile health apps.

Multimedia Appendix 2 User version of Mobile App Rating Scale scoring of the dementia-related mHealth apps.

Multimedia Appendix 3 CONSORT-EHEALTH checklist (V 1.6.1).

## Abbreviations

mHealth: mobile health
uMARS: user version of the Mobile Application Rating Scale

## Data Availability

The study data are identified participant data. The data that support the findings of this study will be available beginning 12 months and ending 36 months following the article publication from the corresponding author (WFM) upon reasonable request.
