# Supplementary material for: Attributes, Quality, and Downloads of Dementia-Related Mobile Apps for Patients With Dementia and Their Caregivers: App Review and Evaluation Study
Source: JMIR Form Res. 2024 Apr 29;8:e51076. doi: 10.2196/51076 (PMC11091808; doi:10.2196/51076)
Supplement: Multimedia Appendix 1 [file formative_v8i1e51076_app1.docx]

## Multimedia Appendix 1

Multimedia Appendix 1. Description and end comments of dementia-related mHealth apps and their app type.

| App name | Downloads | Country | Released year | Platform | App Type |
| --- | --- | --- | --- | --- | --- |
| 1. Memorado   Brain Games | >1,000,000 | Germany | 2015 | Android | standalone digital game therapeutics |
| 1. NeuroNation –   Brain Training & Brain Games | >10,000,000 | Germany | 2014 | Android | standalone digital game therapeutics |
| 3. Brain Track | >5,000 | Australia | 2022 | Android-iOS | standalone digital game therapeutics |
| 4. Daily Brain Snack | >5,000 | Taiwan | 2018 | Android-iOS | standalone digital game therapeutics |
| 5. KettleMind Competitive Brain Games | >100,000 | India | 2018 | Android | standalone digital game therapeutics |
| 6. The Dementia Friendly Home | >500 | Australia | 2016 | Android-iOS | improve clinical outcomes ^a^ |
| 7. CLEAR Dementia Care | >1,000 | U.K. | 2021 | Android-iOS | deliver disease related education |
| 8. Use your Brain Brave | >100 | HongKong | 2020 | Android-iOS | improve clinical outcomes ^a^ |
| 9. Love Long term Care | >10,000 | Taiwan | 2017 | Android | improve clinical outcomes ^a^ |
| 10. Midland Cognitive Assessment | >1,000 | Canada | 2019 | Android | support clinical diagnosis or decision making |
| 11. DemKonnect -Dementia Care App | >500 | India | 2018 | Android | improve clinical outcomes ^a^ |
| 12. Dementia Support | >50 | United States | 2021 | Android-iOS | improve clinical outcomes ^a^ |
| 13. Dementia Talk | >500 | United States | 2019 | Android-iOS | improve clinical outcomes ^a^ |
| 14. Soca Dementia Test | >1,000 | United States | 2018 | Android | support clinical diagnosis or decision making |
| 15. Alz Test | >1,000 | United States | 2020 | Android-iOS | support clinical diagnosis or decision making |
| 16. American Caregiver Association | >100 | United States | 2018 | Android | improve clinical outcomes ^a^ |
| 17. Dementia and Me | >10 | U.K. | 2020 | Android | improve clinical outcomes ^a^ |

^a^ improve clinical outcomes from established treatment pathways through behavior change and enhancement of patient adherence and compliance with treatment

| App name | End comments |
| --- | --- |
| 1. Memorado Brain Games | 1. The application assesses responsiveness, memory, speed, concentration, logical reasoning, and is well-designed in terms of color schemes and functionality.  2. Training with the application is less straightforward for dementia patients and requires some practice over a period of time. |
| 2. NeuroNation -Brain Training & Brain Games | 1. The overall color design and functionality of the application are excellent, with animated videos providing accurate information.  2. The training in the application, which includes speed, reasoning, attention, and memory exercises, is of moderate difficulty for dementia patients and can be practiced daily. |
| 3. Brain Track | 1. The application has excellent graphics and design.  2. The application's training includes emotion recognition, large fonts, the ability to connect with Dementia Australia, and provides knowledge of strategies beneficial for the brain. |
| 4. Daily Brain Snack | 1. The application is in Mandarin, features music, vivid graphics, and a comprehensive set of functions.  2. Training is divided into cognitive, attention, memory, mathematics, and language categories, with a relatively high level of difficulty for dementia patients |
| 5. KettleMind Competitive Brain Games | 1. The interface has a simple color scheme, and the graphics effectively convey the content.  2. Training is divided into categories such as focus, English, visual, reasoning, problem solving, and memory, with a relatively high level of difficulty suitable for dementia patients. |
| 6. The Dementia Friendly Home | 1. The functionality is excellent but rapid application navigation may induce a sense of dizziness.  2. This application can assist caregivers living with dementia patients as a guide to creating a dementia-friendly environment, to ensure that the chande or modifying a home have a positive impact. |
| 7. CLEAR Dementia Care | 1. The application features vivid colors.  2. The primary function of the application is to understand dementia, behavior, and development. |
| 8. Use your Brain Brave | 1. The application is in Mandarin and features bright colors.  2. The primary purpose of the application is to modify the behavior of individuals with mild cognitive impairment by allowing them to perform cognitive exercises at home through the app's videos. |
| 9. Love Long term Care | 1. The application is in Mandarin and features pink colors.  2. The main purpose is to help caregivers find resources, engage in online chats with experts to discuss caregiving issues, and establish treatment pathways. |
| 10. Midland Cognitive Assessment | 1. The application's assessment feature is comprehensive but only takes 15 minutes, serving as a supplementary tool.  2. The primary purpose of the application is to allow users to administer cognitive assessment. |
| 11. DemKonnect -Dementia Care App | 1. Simple color schemes, precise icons.  2. Users can share their experiences in caring for dementia patients within the application. It also allows users to establish treatment pathways by keeping track of appointments made with dementia experts and ensuring compliance with the treatment. |
| 12. Dementia Support | 1. Vibrant color schemes, precise icons.  2. The application primarily serves as a support system for individuals with dementia and offers the capability to connect with an experienced dementia consultant. |
| 13. Dementia Talk | 1. The mobile health app that helps you to change behavior at home.  2. The functions include a behavior tracker, behavior care plan, and caregiving corner. |
| 14. Soca Dementia Test | 1. The application interface is simple, but the text is too small.  2. Dementia early detection through cognitive assessment. |
| 15. Alz Test | 1. The application has a simple color scheme.  2. Several signs of memory impairment were detected. |
| 16. American Caregiver Association | 1. The application provides a lot of information but lacks images.  2. It can support both local and national initiatives for seniors, regardless of whether they are in assisted living facilities or receiving in-home care. |
| 17. Dementia and Me | 1.The application's functionality is insufficient.  2. Its primary function is to record sleep and walking patterns, aiming to facilitate behavior change and enhance patient adherence. |
