# Supplementary material for: Attributes, Quality, and Downloads of Dementia-Related Mobile Apps for Patients With Dementia and Their Caregivers: App Review and Evaluation Study
Source: JMIR Form Res. 2024 Apr 29;8:e51076. doi: 10.2196/51076 (PMC11091808; doi:10.2196/51076)
Supplement: Multimedia Appendix 2 [file formative_v8i1e51076_app2.docx]

**Multimedia Appendix 2**

Multimedia Appendix 2. User version of Mobile App Rating Scale scoring of the dementia-related mHealth apps.

| Value, median (SD) | | | | | | | |
| --- | --- | --- | --- | --- | --- | --- | --- |
| App name | App Quality | Engagement | Functionality | Aesthetics | Information | Subjective | Perceived Impact |
| Total | 3.35 (0.56) | 3.04 (0.82) | 3.76 (0.38) | 3.45 (0.65) | 3.14 (0.88) | 2.77 (0.82) | 3.09 (0.7) |
| 1.Memorado Brain Games | 4.07 (0.46) | 4.4 (0.37) | 4.3 (0.48) | 4.2 (0.87) | 3.4 (1.97) | 4.05 (0.78) | 3.77 (0.87) |
| 2.NeuroNation -Brain Training & Brain Games | 4.04 (0.21) | 4.24 (0.68) | 3.75 (0.31) | 4.13 (0.87) | 4.05 (0.6) | 3.8 (0.76) | 3.73 (0.92) |
| 3.Brain Track | 4.01 (0.13) | 4  (0.57) | 3.9 (0.22) | 4.2 (0.38) | 3.95 (0.37) | 3.7 (0.65) | 3.8 (0.66) |
| 4.Daily Brain Snack | 3.92 (0.9) | 4.08 (0.23) | 4.45 (0.37) | 4.53 (0.38) | 2.6 (1.93) | 4 (1.02) | 2.83 (1.24) |
| 5.KettleMind Competitive Brain Games | 3.87 (0.49) | 4.08 (0.54) | 4.07 (0.37) | 3.15 (0.72) | 3.85 (1.82) | 3.85 (0.42) | 2.77 (1.08) |
| 6.The Dementia Friendly Home | 3.7 (0.24) | 3.36 (0.78) | 3.7 (0.45) | 3.93 (0.68) | 3.8 (0.78) | 2.85 (0.8) | 3.77 (0.96) |
| 7.CLEAR Dementia Care | 3.55 (0.69) | 2.56 (1.02) | 4.1 (0.38) | 3.6 (1.09) | 3.93 (0.48) | 2.3 (1.07) | 3.1 (0.980 |
| 8.Use your Brain Brave | 3.51 (0.67) | 2.56 (0.62) | 4  (0.73) | 3.53 (0.8) | 3.95 (0.65) | 2.45 (0.78) | 3.8 (0.76) |
| 9.Love Long term Care | 3.37 (0.35) | 2.88 (1.01) | 3.7 (0.89) | 3.47 (1.12) | 3.45 (0.82) | 2.85 (1.13) | 2.97 (1.01) |
| 10.Midland Cognitive Assessment | 3.24 (0.49) | 2.84 (0.57) | 3.8 (0.27) | 2.8 (0.51) | 3.5  (0.4) | 2.5 (0.47) | 3.73 (0.22) |
| 11.DemKonnect -Dementia Care App | 3.22 (0.3) | 2.88 (1.01) | 3.45 (0.67) | 3.07 (0.64) | 3.5 (0.59) | 2.6 (0.8) | 3.67 (0.94) |
| 12.Dementia Support | 3.15 (0.77) | 2.24 (0.74) | 3.6 (0.29) | 2.8 (0.51) | 3.95 (0.65) | 2.05 (0.78) | 3.03 (1.31) |
| 13.Dementia Talk | 2.99 (0.82) | 2.8 (0.68) | 3.65 (0.52) | 3.6 (0.55) | 1.9 (1.76) | 2.1 (1.01) | 2.7 (1.11) |
| 14.Soca Dementia Test | 2.78 (0.62) | 2.44 (0.73) | 3.7 (0.89) | 2.53 (1.17) | 2.43 (1.82) | 2.35 (0.7) | 2.73 (1.19) |
| 15.Alz Test | 2.63 (0.55) | 2.32 (0.36) | 3.4  (0.7) | 2.67 (0.85) | 2.15 (1.29) | 2.25 (1.02) | 2.17 (1.24) |
| 16.American Caregiver Association | 2.63 (0.45) | 2.04 (0.52) | 3.15 (1.17) | 2.67 (0.62) | 2.65 (1.69) | 2.1 (1.33) | 2.63 (1.28) |
| 17.Dementia and Me | 2.25 (0.95) | 2  (0.14) | 3.05 (0.89) | 2.93 (0.72) | 1  (1.02) | 1.3 (0.41) | 1.27 (0.6) |
